# The structure of His15 acetamide-modified hen egg-white lysozyme: a nice surprise from an old friend

**DOI:** 10.1107/S2053230X2500010X

**Published:** 2025-01-13

**Authors:** Jose Malanho da Silva, Jose Lanuza, Francesco Bruno, Vito Calderone, Enrico Ravera

**Affiliations:** a Department of Chemistry ‘Ugo Schiff’ Università degli Studi di Firenze Via della Lastruccia 3 50019Sesto Fiorentino Italy; b Magnetic Resonance Center (CERM) Università degli Studi di Firenze Via Luigi Sacconi 6 50019Sesto Fiorentino Italy; c Consorzio Interuniversitario Di Risonanze Magnetiche Di Metalloproteine Paramagnetiche Via Luigi Sacconi 6 50019Sesto Fiorentino Italy; Centro Nacional de Biotecnología – CSIC, Spain

**Keywords:** acylated lysozyme, crystallization, space-group analysis, His15 acetamide modification

## Abstract

Hen egg-white lysozyme (HEWL) is a small polycationic protein which is highly soluble and stable. This has led to it becoming a ‘molecular laboratory’ where chemical biological operations and structural techniques are tested. To date, HEWL accounts for 1233 PDB entries, roughly 0.5% of the total, making it the best-represented protein in the PDB. With the aim of unambiguously identifying the N atom of the His15 side chain that is most reactive towards iodoacetamide, the structure of chemically modified HEWL was determined by crystallizing it using the ‘15 minutes lysozyme’ protocol. This protocol invariably yields tetragonal crystals of the unmodified protein. To our surprise, we found that the crystals of the modified protein had similar unit-cell parameters but that refinement was only possible when considering an orthorhombic system.

## Abstract 2

Chemical modification of lysozyme at His15 using iodoacetamide was investigated structurally. Crystallization of the chemically modified enzyme using a protocol which yields tetragonal crystals gave orthorhombic crystals with similar unit-cell parameters.

## 1. Introduction

Hen egg-white lysozyme (HEWL) is the best-represented structure in the PDB. It was one of the earliest proteins to be crystallized and its structure was among the first to be solved using X-ray crystallography (Blake *et al.*, 1965 ▸, 2012 ▸; Johnson, 1966 ▸). There are several reasons why lysozyme is so prevalent in the PDB (Goodsell, 2000 ▸).

*Ease of crystallization*. HEWL is relatively easy to crystallize, making it a popular model for structural biology studies.

*Historical significance*. HEWL was one of the first enzymes for which the 3D structure was solved, which led to its extensive use as a model system for the study of protein structure and crystallization techniques.

*Wide application*. HEWL is used as a model protein in various experimental studies related to enzyme function, protein folding and molecular dynamics.

HEWL is often defined as a ‘molecular laboratory’ because its solubility and outstanding stability make it amenable to several chemical biological manipulations while still being able to crystallize (Strynadka & James, 1996 ▸; Helliwell *et al.*, 1996 ▸, 2010 ▸; Tanley *et al.*, 2014 ▸, 2016 ▸; Mitchell *et al.*, 2023 ▸; Helliwell & Tanley, 2016 ▸; Brink & Helliwell, 2019 ▸). Not surprisingly, therefore, it was the test case for the pioneering spin-labelling experiment presented by Harden McConnell and coworkers in 1972 (Wien *et al.*, 1972 ▸). We will come back to this below.

In 2006, Luckarift and coworkers demonstrated that HEWL can promote the formation of silica and titania from soluble precursors, similar to the function performed in silica by natural polycationic molecules found in diatoms (Luckarift *et al.*, 2006 ▸). The nature of the interactions between HEWL and the precursor, and between HEWL and the formed material, have been investigated by several groups, and are still a matter of debate (see below). The results that we have obtained in this research field are summarized in the following paragraph.

A tetrahedral precursor species for titania was found by X-ray crystallography to interact with a positively charged patch in the vicinity of Arg13 (Gigli *et al.*, 2021 ▸). The interaction appears to be mediated by intervening water molecules. The same experiment could not be completed with the precursor of silica (silicic acid) because the polycondensation is so rapid that soaking the crystals is impractical. Therefore, we resorted to molecular-dynamics simulations, resorting to a nonreactive force field so that it was possible to define interactions before the precursor molecule reacted. In this way, we found that the accumulation of silicic acid occurs at positively charged patches and, again, in the vicinity of Arg13 (Macchiagodena *et al.*, 2024 ▸). Above, we wrote that the behaviour of lysozyme is ‘similar’ to that of natural peptides and other polycations. However, it has been postulated by Lenoci & Camp (2006 ▸, 2008 ▸) that natural polycations undergo some kind of phase separation before silica can grow at the interface between the two phases (Sumper, 2004 ▸; Sumper & Brunner, 2006 ▸; Nassif & Livage, 2011 ▸). This hypothesis has very recently found brilliant experimental verification (Zhai *et al.*, 2022 ▸, 2023 ▸; Strobl *et al.*, 2023 ▸; Kozak *et al.*, 2024 ▸). What, then, did we mean previously by ‘similar’? This investigation is still ongoing through the MInO project (https://mino.cerm.unifi.it/), and requires a very robust structural biology platform, which is currently under development in our laboratory.

We then turned our attention to the final composite material consisting of the bioinspired inorganic oxide and, potentially, the protein template. Previous work suggested that the protein underwent partial unfolding and, at the end of the process, was excluded from the composite (van den Heuvel *et al.*, 2018 ▸; Stawski *et al.*, 2019 ▸). Our experimental observations, relying upon solid-state NMR, small-angle scattering, microscopy and biochemical tests, suggest that lysozyme is not denatured and is sterically trapped within the condensed network of the silica, at least for 80% of its mass in the composite. The remaining 20% indeed interacts electrostatically (Bruno *et al.*, 2022 ▸). This leads us back to the McConnell’s seminal 1972 paper on spin-labelling (Wien *et al.*, 1972 ▸). We performed the same spin-labelling reaction to be able to monitor the motion of HEWL inside the composite by EPR spectroscopy as had been performed for the same protein but different composites by Antonov *et al.* (2020 ▸). What we found was that even if the electrostatic interaction between the protein and the silica does not completely account for the fact that HEWL is trapped within the composite, it still dictates an orientational preference, according to which the protein sits with its active site pointing towards the surface in about 60% of occurrences (Bruno *et al.*, 2023 ▸). This is also in line with the observation of antibacterial activity of the composite reported by Luckarift *et al.* (2006 ▸). In order to extend atomistic simulations to this situation, we need a proper model for the chemically modified side chain. Therefore, we looked for a structure-based verification of the hypothesis by Wien *et al.* (1972 ▸) that it is the N^ɛ^ atom of the His15 side chain which acts as a nucleophile on the iodo­acetamide moiety in the spin label. We thus performed the reaction of HEWL with iodoacetamide and obtained the structure of this chemically modified HEWL.

## 2. Materials and methods

HEWL and iodoacetamide were purchased from Sigma–Aldrich (Merk Life Science S.r.l., Milano, Italy) and were used without any further purification.

The reaction of iodoacetamide and HEWL was performed as follows: 70 mg of HEWL and 14 mg of iodoacetamide were dissolved in 1 ml 0.1 *M* sodium acetate pH 5.1 and the mixture was shaken at 300 rev min^−1^ for 70 h at 40°C using an MB-102 mixing block (Bioer, People’s Republic of China). After the reaction, the protein was washed with the same buffer, without iodoacetamide, in a centrifugal concentrator at 10 000 rev min^−1^ for 15 min with Millipore Amicon Ultra 0.5 ml (regenerated cellulose membrane, 3 kDa MWCO; Merk Life Science S.r.l., Milano, Italy). The washing process involved concentrating and then diluting the protein with a dilution factor of ten, and was performed ten times to ensure elimination of unreacted iodoacetamide. The efficiency of the tagging reaction was estimated to be 50% on the basis of the NMR spectrum (Goux & Allerhand, 1979 ▸); see Supplementary Fig. S1, in which quantification performed with *TrAGICo* (https://github.com/letiziafiorucci/tragico) and with *Klassez* (https://github.com/MetallerTM/klassez) is also reported. At this point, the sample was immediately used for crystallization.

Crystals of iodoacetamide-reacted HEWL (AM-HEWL) were obtained in sitting drops by adding a 2 µl aliquot of protein solution (0.1 *M* sodium acetate pH 5.1, 8 mg ml^−1^ HEWL pH 5.1) to 2 µl reservoir buffer [0.05 *M* sodium acetate, 30%(*w*/*v*) PEG MME 5000, 1.0 *M* sodium chloride pH 4.6] and storing at 20°C. Crystallization trials were set up manually in 24-well Cryschem plates (Hampton Research, USA) using a reservoir volume of 1 ml. Crystals appeared in a few hours.

The data set was collected in-house using a Bruker D8 Venture copper-anode diffractometer equipped with a PHOTON II detector at 100 K using Cu *K*α radiation corresponding to a wavelength of 1.54 Å; the crystal used for data collection was used without cryoprotection since it was already cryoprotected by the crystallization conditions. The exposure time was 20 s per frame with an oscillation range of 0.5°, and a total of 720 images were collected. The crystal diffracted to 1.6 Å resolution; it belonged to space group *P*2_1_2_1_2_1_ with two molecules in the asymmetric unit, a solvent content of about 50% and a mosaicity of 0.3° (see below). The data were processed using *XDS* (Kabsch, 2010 ▸), reduced and scaled using *XSCALE* (Kabsch, 2010 ▸) and amplitudes were calculated using *XDSCONV* (Kabsch, 2010 ▸). The structure was solved using the molecular-replacement technique and showed the presence of two molecules in the asymmetric unit; the starting model used was PDB entry 7a70 (Gigli *et al.*, 2021 ▸). The successful orientation and translation of the molecule within the crystallographic unit cell was determined with *MOLREP* (Vagin & Teplyakov, 2000 ▸). Refinement and water-position assignment were carried out using *Phenix* (Liebschner *et al.*, 2019 ▸), applying TLS restraints and using anisotropic *B* factors for sodium and chlorine only. Between the refinement cycles, the model was subjected to manual rebuilding using *Coot* (Emsley *et al.*, 2010 ▸). The quality of the refined structure was assessed using *MolProbity* (Chen *et al.*, 2010 ▸).

The data were also indexed in the tetragonal space group *P*4_3_2_1_2 (see Section 3).

Data-processing and refinement statistics for both space groups are shown in Table 1 ▸. Coordinates and structure factors have been deposited in the PDB with accession code 9gyh.

## 3. Results and discussion

**Figure 1:**
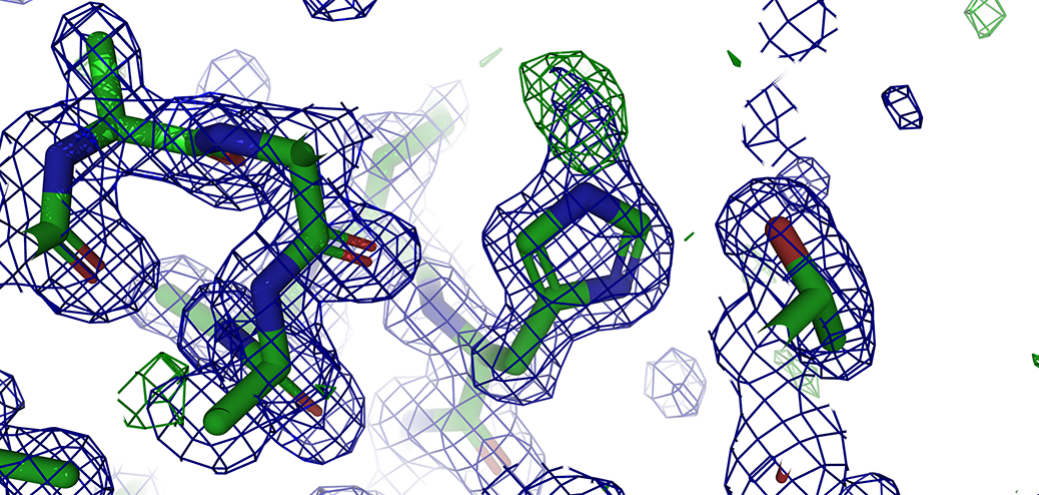
*PyMOL* ray-traced representation of the 2*F*_o_ − *F*_c_ electron-density map contoured at the 1.0σ level (blue) and of the *F*_o_ − *F*_c_ electron-density map contoured at the 3.0σ level (green) showing the additional electron density around N^ɛ^ of His15.

The overall structure of the protein is superimposable with several lysozyme structures present in the PDB (for example, the r.m.s.d. to PDB entry 1iee is 0.274 Å), which confirms the relative lack of effect of the chemical modification of a solvent-exposed histidine side chain. The presence of the derivatization of His15 is apparent (Fig. 1 ▸), and confirms the proposal of Wien *et al.* (1972 ▸) that the reaction occurs at N^ɛ^. This is consistent with the different reactivity that can be predicted for the two N atoms: for N^δ^ the p*K*_a_ can be estimated to be around 9, whereas that for N^ɛ^ has been determined to be around 5.5 ± 0.2 (Webb *et al.*, 2011 ▸), which means that under the reaction conditions N^ɛ^ is the most likely donor for the SN_2_ reaction.

**Figure 2:**
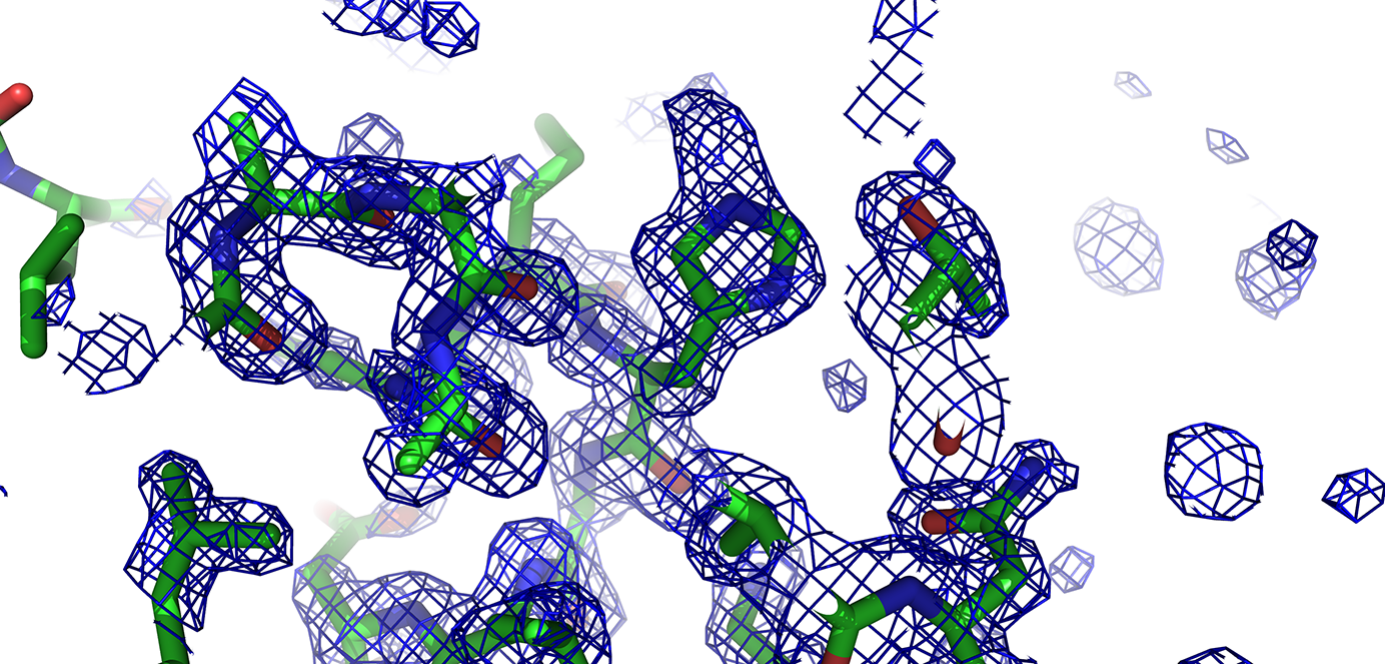
*PyMOL* ray-traced 2*F*_o_ − *F*_c_ composite omit map contoured at 1.0σ confirming the additional electron density around N^ɛ^.

As a further confirmation of the presence of the derivatization, a composite omit map was calculated with *Phenix* on a modified coordinate file where the derivatization was absent from both histidine residues; extra density is clearly visible on N^ɛ^ (Fig. 2 ▸).

**Figure 3:**
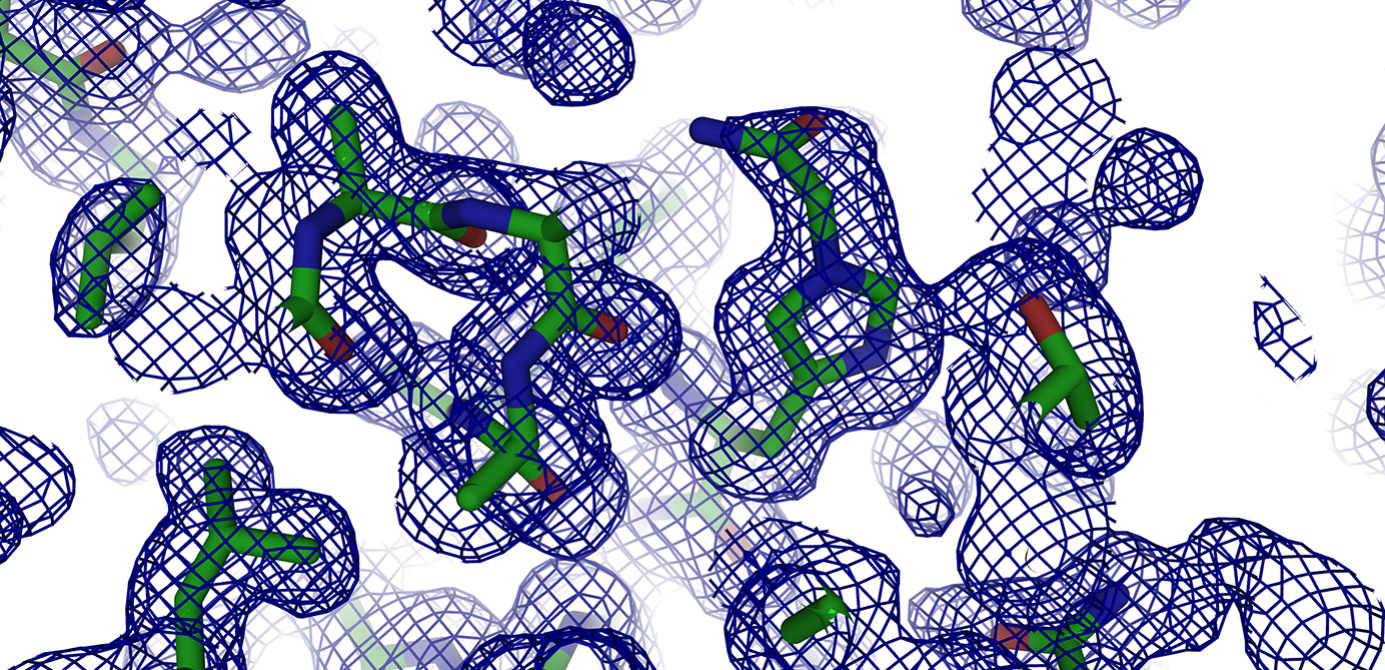
*PyMOL* ray-traced electron-density map contoured at 0.3σ showing basically complete electron density around the acetamide moiety at N^ɛ^ of His15.

The reaction site is water-exposed, and the density of the acetamide moiety appears to be lower than the density of the His15 side chain. This indicates free rotation about the N^ɛ^—C_2_ and C_1_—C_2_ single bonds, which is consistent with the relatively large mobility of the spin label observed by Wien *et al.* (1972 ▸). Nevertheless, if the 2*F*_o_ − *F*_c_ map is contoured at values around 0.5–0.6σ, density for the full acetamide moiety becomes apparent (Fig. 3 ▸) and the C_2_ atom can be refined at full occupancy: the *B* factor of N^ɛ^ is around 23 Å^2^ in both molecules in the asymmetric unit, and that of the acyl C_2_ carbon directly attached to the imidazole ring is 37 and 30 Å^2^ in molecules *A* and *B*, respectively. These values are in line with those of the surrounding water molecules. The higher *B* factor for the acyl C_2_ carbon in molecule *A* can also be related to a lower occupancy (around 0.8).

The experimental structure provides a direct observation and hence an unambiguous verification of the reaction at N^ɛ^. Whereas this might appear to be a merely confirmatory result, we wish to stress that this is the first experimental structure showing this kind of chemical modification of the histidine side chain.

This crystal structure also has a rather unexpected feature with respect to the conditions that it was obtained in: the crystallization conditions used in this work (‘15 minutes lysozyme’) are some of the most standard conditions used for lysozyme and have been used in (for example) serial experiments (Casanas *et al.*, 2016 ▸; Leonarski *et al.*, 2018 ▸); the space group obtained under such conditions is invariably tetragonal *P*4_3_2_1_2. Surprisingly, the space group in this case was ortho­rhombic *P*2_1_2_1_2_1_. The unit-cell parameters are very similar to those for the expected tetragonal case, but the *b* and *c* axes of the cell diverge significantly (78.17 versus 79.31 Å), which makes it impossible to process the data as tetragonal with good statistics. Table 2 ▸ shows the possible space groups as determined by *XDS* during data processing: it is apparent that the expected tetragonal space group has a very high penalty with respect to the orthorhombic space group with the same axis. For this reason, the data have been processed, solved and refined in these two space groups and, again, the comparison suggests that the plausible space group is the orthorhombic space group (Table 1 ▸). This hypothesis is further supported by the outcome of a refinement performed using strict NCS restraints (with the default torsion-angle protocol in *Phenix*) between the two independent molecules in the asymmetric unit of the orthorhombic cell. Imposing the NCS restraint does not affect the *R*_free_ and *R*_cryst_ values at all (see Supplementary Table S1). This implies that the difference between the orthorhombic and tetragonal space groups is not linked to the difference (in for example occupancy) between the two molecules.

The direct consequence of such a space-group change is the number of independent molecules in the asymmetric unit. In the case of the tetragonal space group there is only one molecule in the asymmetric unit, whereas of course the number of molecules becomes two in the case of an ortho­rhombic space group with similar unit-cell parameters.

The orthorhombic space group is not an absolute novelty for HEWL since about 70 orthorhombic entries for this protein are already present in the PDB (Schirò *et al.*, 2020 ▸), with the highest resolution example being PDB entry 6f1o (Plaza-Garrido *et al.*, 2018 ▸). What appears to be unique, however, are the unit-cell parameters of the orthorhombic space group in the present work, which are basically equivalent to those in the tetragonal case. In contrast, in other orthorhombic lysozyme structures the unit-cell parameters are around *a* = 30, *b* = 55–57, *c* = 66–73 Å.

**Figure 4:**
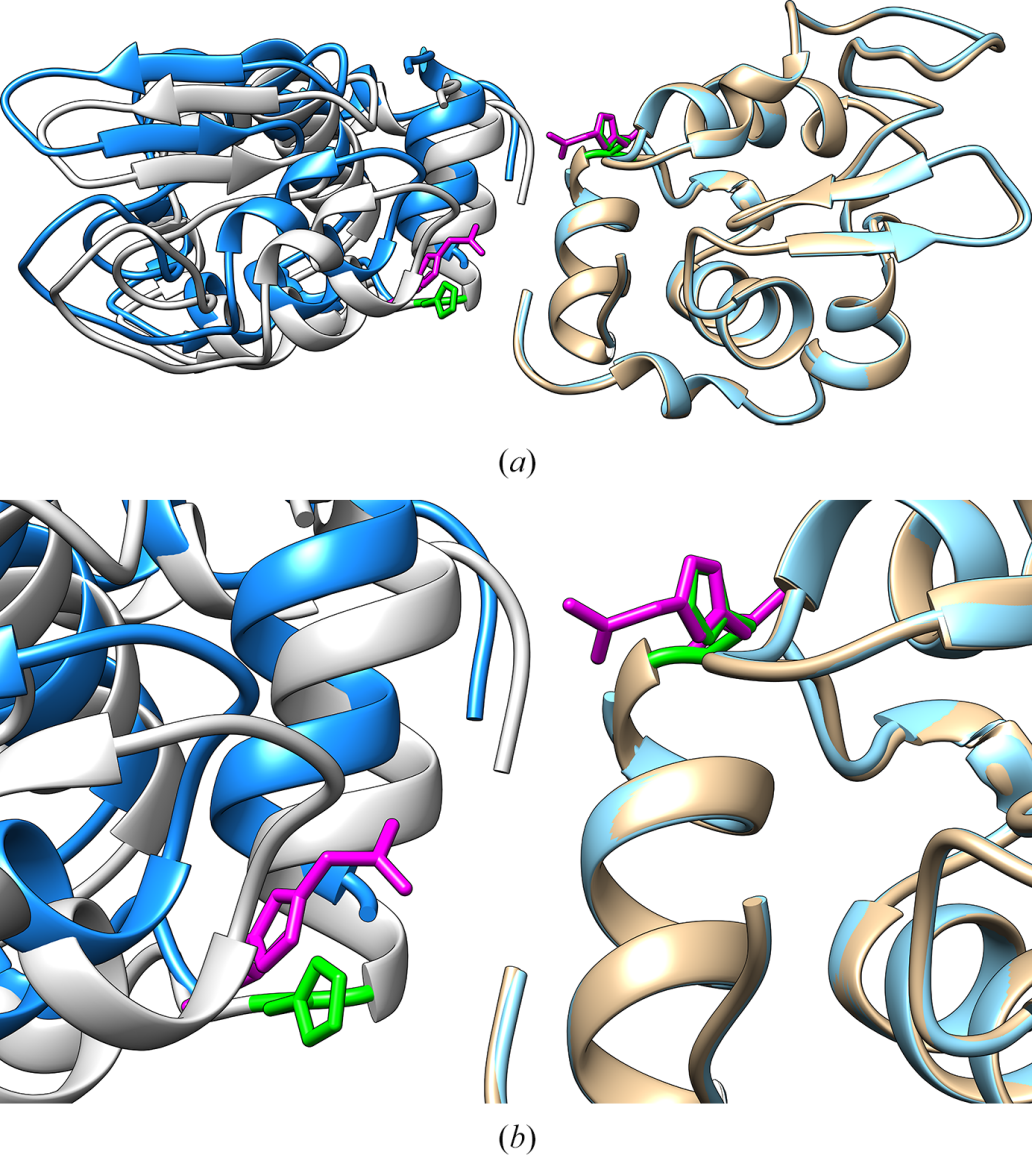
Overlay obtained with *UCSF Chimera* (Pettersen *et al.*, 2004 ▸) of two crystal copies for the tetragonal PDB entry 1iee with His15 in green and chain *A* of PDB entry 9gyh with the modified His15 in magenta.

This particular behaviour can be attributed, at least in part, to the following aspect of the tetragonal lysozyme crystals: taking the tetragonal structure at the highest available resolution (PDB entry 1iee) as a reference (Sauter *et al.*, 2001 ▸), His15 is located at the interface between two molecules in the crystal. The additional hindrance added by the, albeit flexible, acetamide moiety disturbs this particular crystal contact (Fig. 4 ▸), pushing back the contacting molecule by a few ångströms. Of course it is still necessary to take an incomplete chemical modification into account, which could lead to the presence of two independent molecules in the asymmetric unit.

## 4. Conclusions

While looking for a structure-based verification of the statement found in the pioneering spin-labelling paper by Wien *et al.* (1972 ▸) that iodoacetamide reacts with His15 at N^ɛ^, we discovered that this chemical modification induces a very unusual crystal structure in HEWL. Derivatization at His15 N^ɛ^ is clearly supported by the electron-density map, and the observed heterogeneity about the freely rotatable bonds of newly formed His–acetamide side chains is consistent with previous EPR observations. However, the crystallographic findings go beyond identifying the reaction site, as HEWL crystallized in the orthorhombic space group *P*2_1_2_1_2_1_, an uncommon observation under the well established conditions used, which invariably yield crystals in the tetragonal space group *P*4_3_2_1_2. The uncommon finding is not so much the space group itself, which has some tens of entries in the PDB, but rather that the unit-cell parameters of such a space group are very similar to those of the tetragonal space group. This finding shows how even minor modifications can impact on protein crystallization, even in systems that have been as extensively characterized as HEWL.

## Supplementary Material

PDB reference: hen egg-white lysozyme, His15 functionalized with iodoacetamide, 9gyh

Supplementary Table and Figure. DOI: 10.1107/S2053230X2500010X/va5062sup1.pdf

## Figures and Tables

**Table 1:** Data-processing and refinement statistics for both the orthorhombic and the putative tetragonal space groups Values in parentheses are for the highest resolution shell.

	Orthorhombic	Tetragonal
Wavelength (Å)	1.541	1.541
Resolution range (Å)	21.78–1.60 (1.657–1.600)	22.33–1.60 (1.700–1.600)
Space group	*P*2_1_2_1_2_1_	*P*4_3_2_1_2
*a*, *b*, *c* (Å)	37.53, 78.17, 79.31	78.61, 78.61, 37.49
α, β, γ (°)	90, 90, 90	90, 90, 90
Total reflections	219014 (12680)	212818 (22591)
Unique reflections	31328 (2966)	16009 (2614)
Multiplicity	7.0 (4.3)	13.3 (8.6)
Completeness (%)	99.28 (95.65)	99.81 (99.92)
Mean *I*/σ(*I*)	14.37 (2.77)	8.00 (2.71)
Wilson *B* factor (Å^2^)	11.04	9.78
*R*_merge_	0.09773 (0.6069)	0.2158 (0.8032)
*R*_meas_	0.1052 (0.6887)	0.2242 (0.8533)
*R*_p.i.m._	0.03792 (0.3200)	0.05929 (0.2844)
CC_1/2_	0.998 (0.801)	0.991 (0.797)
CC*	0.999 (0.943)	0.998 (0.942)
Reflections used in refinement	31327 (2966)	16009 (2614)
Reflections used for *R*_free_	1567 (148)	801 (131)
*R*_work_	0.1728 (0.2309)	0.2095 (0.2396)
*R*_free_	0.2086 (0.2889)	0.2430 (0.2860)
No. of non-H atoms		
Total	2420	1138
Macromolecules	1982	991
Ligands	38	19
Solvent	400	128
Protein residues	256	128
R.m.s.d., bond lengths (Å)	0.010	0.010
R.m.s.d., angles (°)	1.05	1.08
Ramachandran favoured (%)	97.98	97.58
Ramachandran allowed (%)	2.02	2.42
Ramachandran outliers (%)	0.00	0.00
Rotamer outliers (%)	0.48	0.96
Clashscore	3.83	6.63
Average *B* factor (Å^2^)		
Overall	16.04	13.76
Macromolecules	14.46	12.88
Ligands	24.49	25.10
Solvent	23.07	18.89

**Table 2:** Log from the *XDS* indexing routine showing the penalty for the tetragonal space group (tP) with respect to the orthorhombic space group (oP)

Lattice character	Bravais lattice	Quality of fit	*a* (Å)	*b* (Å)	*c* (Å)	α (°)	β (°)	γ (°)
44	aP	0.0	37.6	78.2	79.3	90.1	90.0	90.0
31	aP	0.3	37.6	78.2	79.3	89.9	90.0	90.0
35	mP	1.0	78.2	37.6	79.3	90.0	90.1	90.0
34	mP	2.5	37.6	79.3	78.2	90.1	90.0	90.0
33	mP	2.9	37.6	78.2	79.3	90.1	90.0	90.0
32	oP	3.2	37.6	78.2	79.3	90.1	90.0	90.0
25	mC	61.7	111.4	111.5	37.6	90.0	90.0	89.2
23	oC	62.2	111.4	111.5	37.6	90.0	90.0	89.2
20	mC	62.4	111.5	111.4	37.6	90.0	90.0	90.8
21	tP	64.4	78.2	79.3	37.6	90.0	90.0	90.1
39	mC	249.5	160.9	37.6	79.3	90.0	90.1	76.5
37	mC	250.5	163.1	37.6	78.2	90.0	90.1	76.7
38	oC	251.8	37.6	160.9	79.3	89.9	90.0	103.5
