# Supplementary material for: The structure of His15 acetamide-modified hen egg-white lysozyme: a nice surprise from an old friend
Source: Acta Crystallogr F Struct Biol Commun. 2025 Jan 13;81(Pt 2):41–6. doi: 10.1107/S2053230X2500010X (PMC11783178; doi:10.1107/S2053230X2500010X)
Supplement: Supplementary file 1 [file f-81-00041-sup1.pdf]

Acta Cryst F

STRUCTURAL BIOLOGY COMMUNICATIONS

Volume 81 (2025)

Supporting information for article:

The structure of His15 acetamide-modified hen egg-white lysozyme: a nice surprise from an old friend

Jose Malanho da Silva, Jose Lanuza, Francesco Bruno, Vito Calderone and Enrico Ravera

S1. Orthorhombic refinement imposing NCS

The refinement in the P 21 21 21 space group was repeated imposing NCS restraints. The refinement statistics are given in table S1.

Table S1 Refinement statistics for orthorhombic space group taking NCS restraints into account

	Orthorhombic with NCS
Reflections used in refinement [1]	31327 (1365)
Reflections used for R-free	1567 (68)
R-work	0.1743 (0.2373)
R-free	0.2100 (0.2870)
Number of non-hydrogen atoms	2420
macromolecules	1982
ligands	38
solvent	400
Protein residues	256
RMS(bonds)	0.006
RMS(angles)	0.81
Ramachandran favored (%)	98.39
Ramachandran allowed (%)	1.61
Ramachandran outliers (%)	0.00
Rotamer outliers (%)	0.48
Clashscore	2.30
Average B-factor	16.06
macromolecules	14.33
ligands	24.12
solvent	23.88

[1]Statistics for the highest-resolution shell are shown in parentheses.

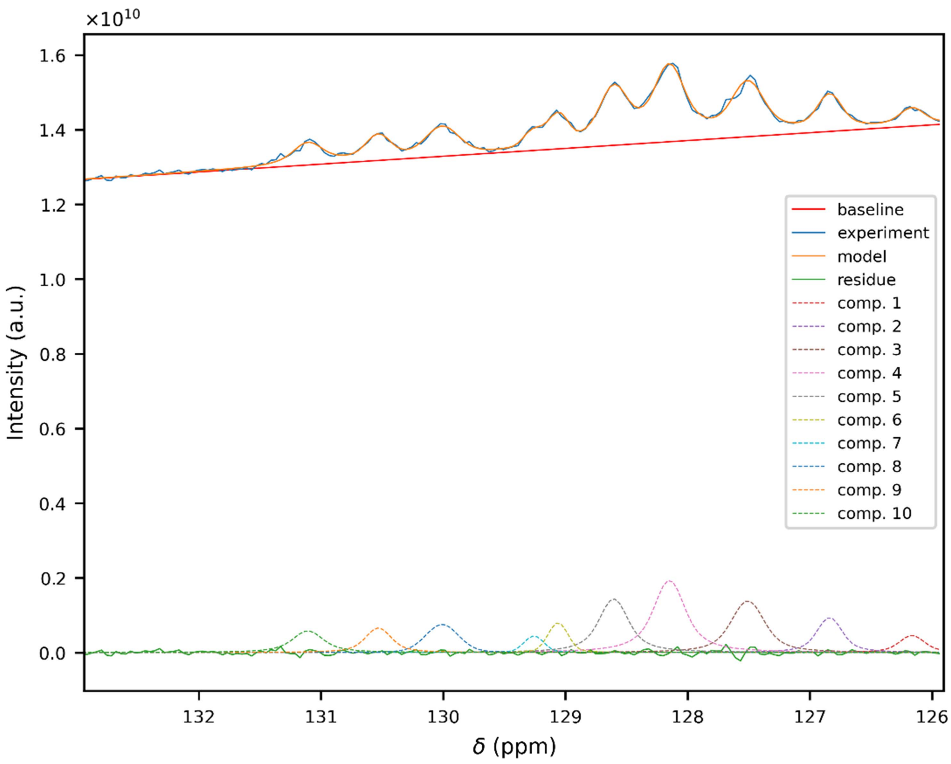

Figure S1 Region of the 13C NMR spectrum where the signal of H15 Cγ is observed according to (Goux & Allerhand, 1979). The spectrum has been acquired at 176 MHz ^{13}C Larmor frequency on a Bruker NEO spectrometer, equipped with a CPTXO probe. The fit of the spectrum was performed without baseline subtraction, as the baseline is automatically accounted for in TrAGICo. The peak corresponding to the Cγ of the free and the functionalized H15, according to the assignment by (Goux & Allerhand, 1979) are indicated as component 8 and component 10 in the fit. The relative intensity of the two species is 1:1.
